# Road Traffic Accidents in Sindhudurg District, Maharashtra: A Spatiotemporal Analysis

**DOI:** 10.7759/cureus.84872

**Published:** 2025-05-27

**Authors:** Neha V Kamble, Dattaprasad Sawant, Elton Fernandes

**Affiliations:** 1 Community Medicine, Grant Government Medical College, Mumbai, IND; 2 Community Medicine, Rajiv Gandhi Medical College, Thane, IND; 3 Community Medicine, Seth Gordhandas Sunderdas (GS) Medical College and King Edward Memorial (KEM) Hospital, Mumbai, IND; 4 Community Medicine, Ram Krishna Medical College Hospital and Research Centre, Bhopal, IND; 5 Community Medicine, KJ Somaiya Medical College and Research Centre, Mumbai, IND

**Keywords:** gis in health, hotspot analysis, public health policy, road traffic injury, spatiotemporal analysis

## Abstract

Introduction

Road traffic accidents (RTAs) are a major global public health issue. Despite being Maharashtra's least populous district, Sindhudurg reports a gradual increase in traffic crashes. This study aims to analyze the spatiotemporal distribution of traffic crashes in Sindhudurg and identify crash hotspots with high-incidence locations.

Methodology

This observational study used secondary data from a local news agency, reporting RTAs and crashes in 2021. The accident site's coordinates (latitude, longitude, and altitude) were obtained using available details and web-based tools. The data were analyzed with Quantum Geographic Information System (QGIS) v3.28 (QGIS Development Team, Johannesburg, South Africa) and ArcMap v10.8 (Esri, Redlands, CA, US) to create heatmaps and cluster maps and conduct hotspot analysis, including spatial autocorrelation.

Results

In 2021, Sindhudurg recorded 176 RTAs, resulting in 38 fatalities and 222 injuries, accounting for 0.6% of Maharashtra’s total accidents. Most incidents occurred along NH-66, with heatmaps revealing four high-density crash zones. Hotspot analysis identified two critical zones on NH-66 in northern Sindhudurg, with a concentration of accidents in low-altitude areas.

Conclusion

This study identified significant RTA hotspots in Sindhudurg, using geospatial analysis to highlight high-risk areas. These findings support the development of targeted strategies to reduce crashes, improve road safety, and foster sustainable transportation in India's first tourism district.

## Introduction

In 2021, India recorded 412,432 road traffic accidents (RTAs), resulting in 153,972 fatalities and 384,448 injuries, with a fatality rate of 37.7 deaths per 100 accidents [1]. Two-wheeler riders accounted for the highest fatalities, with 69,240 deaths [1]. RTAs are particularly prevalent on national and state highways. Despite comprising only 2% of India's road network, national highways (NH) and expressways accounted for 36% of road deaths, with a fatality rate of 0.42 deaths per kilometer annually [2]. RTAs impose an economic burden of 3%-5% of the national GDP, and many injuries go unreported, leading to an underestimation of RTAs [3]. Hospitalization cases range between two and three million annually [2].

In 2021, Maharashtra reported 24,477 RTAs, with 13,528 fatalities and 23,071 injuries, resulting in a fatality rate of 55.3 deaths per 100 accidents, higher than the national average [1]. Road crashes exhibit both temporal and spatial variation [4]. Identifying traffic accident patterns and hotspots is essential for enhancing road safety, transportation planning, and policy development [4]. Without adequate safety measures, RTAs could claim an estimated 241,751 lives by 2030, and the mortality rate may continue rising until 2042 without policy changes [5]. RTAs place a significant financial strain on families, particularly in rural areas where victims are often the primary earners [5]. This highlights the urgent need for comprehensive road safety measures.

Sindhudurg district, with an area of 5,207 km^2^ and a population of 868,825, borders the Arabian Sea to the west, Ratnagiri to the north, and Goa to the south [6]. It comprises eight talukas: Devgad, Kankavli, Kudal, Malvan, Sawantwadi, Vaibhavwadi, Dodamarg, and Vengurla, and is well-connected by NH-17 (now NH-66), passing through towns like Kankavli, Kudal, and Sawantwadi [6]. Though one of the least populous districts in Maharashtra, it is a key tourism hub and is traversed by NH-66, a major arterial route that connects Mumbai-Goa. This highway caters to a constant flow of tourists, who are unfamiliar with local road conditions and accident-prone areas, increasing their risk of RTAs. Sindhudurg has reported a 31.67% increase in mortality due to RTAs in 2018 compared to 2016 [7].

Without clear identification of accident hotspots and timing patterns, policymakers and local planners lack the information required to implement targeted interventions. A spatiotemporal analysis of RTAs can support local road safety planning, inform infrastructure improvements, and aid in developing evidence-based policies to reduce accident risk and improve public safety along NH-66. Although India is the second most populous country, research on road traffic injuries remains limited, comprising only 0.7% of global studies [2]. There is a dearth of research on RTAs in Maharashtra, including Sindhudurg. Highways, mountainous terrain, and tourism-related traffic contribute to accident-prone zones in the district. Identifying and analyzing these high-risk areas through spatiotemporal analysis are crucial for targeted interventions.

Geographic Information Systems (GIS) have proven vital for studying RTAs and identifying dangerous areas [7]. While India has made progress with GIS applications, such as the Integrated Road Accident Database (iRAD), its full potential remains underutilized due to data accuracy and real-time update constraints [8]. Many injuries go unreported, leading to an underestimation of RTAs [3]. However, the local newspapers were found to report the RTA location precisely with detailed accident timing and outcomes. Local GIS-based studies in districts like Sindhudurg can bridge this gap and provide policymakers with evidence-based insights.

This study aims to analyze 2021 RTAs in Sindhudurg district through spatiotemporal methods using GIS. The year 2020 saw significant disruptions in mobility due to COVID-19 lockdowns and travel restrictions, which likely led to an abnormal reduction in road traffic and accident occurrences. In contrast, 2021 reflects a more stabilized post-lockdown period with the gradual resumption of travel and tourism activities, particularly along NH-66. As such, data from 2021 provide a more representative baseline for analyzing typical road traffic patterns. These findings can serve as a foundation for future multi-year comparative studies to assess evolving trends and the impact of policy interventions. We seek to identify high-risk zones by mapping accident hotspots and examining trends in accidents across various time periods, including daily, monthly, and seasonal variations.

## Materials and methods

This observational study was based on secondary data of RTAs and traffic crashes in Sindhudurg district, Maharashtra, India. Data on RTAs and traffic crashes were obtained from a news agency (Prahaar; Rane Prakashan Private Limited; https://prahaar.in) from its regional edition of Sindhudurg district. A RTA is defined as an accident occurring on a road or road open to public transport, resulting in the death or injury of one or more individuals and the presence of at least one moving vehicle, whereas a traffic crash is an event involving the movement of at least one road vehicle on a road, which results in death, injury to a person, or property damage [4,9]. All the electronic editions of issues from January 1, 2021, to January 5, 2022, were accessed from the official website of the newspaper (https://epaper.prahaar.in), and all RTAs and traffic crashes reported in the issues were compiled. Details of RTA and crashes such as date, time, location, vehicles involved, and injured and dead persons were obtained and organized using Microsoft Excel version 2021 (Microsoft Corp., Redmond, WA, US). The exact geographical coordinates of the accident sites were determined using available location details in the news and local resources. The extracted geographical coordinates were projected on Google Maps (Google, Mountain View, CA, US; https://www.google.com/maps/) to confirm the location. Additionally, altitude (m) was found with the help of the web-based application Elevation Finder (Free Map Tools; https://www.freemaptools.com/elevation-finder.htm). All the data is converted into a comma-delimited text file to project for geospatial analysis in various software. This study includes secondary data, and no human participants were involved at any stage; hence, ethical clearance was not taken.

Geospatial analysis

The comma-delimited file was projected in Quantum GIS (QGIS) version 3.28 (QGIS Development Team, Johannesburg, South Africa) on World Geodetic System 1984 (WGS84). Spot maps of taluka-wise and village-wise RTAs and crashes were made in QGIS along with different base layers (road network map, satellite map, etc.) and heatmap presentations. ArcMap version 10.8 in Aeronautical Reconnaissance Coverage GIS (ArcGIS) (Esri, Redlands, CA, US) was used to perform advanced spatiotemporal analytical techniques to examine RTAs and crashes in the district. The following variables were used in the spatiotemporal analysis: date of crash as per the Gregorian Calendar, total number of deaths and injured persons, total number of driver fatalities, total number of passenger fatalities and injuries, total number of pedestrian fatalities, total number of pedestrian injuries, etc. The cause of the RTA and crash was not available for all the events, hence excluded from the analysis. Geographically Weighted Regression (GWR), Global Moran’s I, and the Getis-Ord Gi* models were used to evaluate spatial autocorrelation and distribution [4,9]. Global Moran’s I was used to find a significant relationship between the values of variables of a specific location and the values of neighboring locations. Getis-Ord Gi* was used to find hotspot areas of RTAs and crashes. Data were also entered in SPSS version 26 (IBM Corp., Armonk, NY, US) for descriptive analysis where percentages of different variables were calculated. The binomial test and chi-squared test were used to compare different proportions, and the significance level was set at p < 0.05. All the pictorial maps were created using QGIS version 3.28 and ArcMap version 10.8 with standard procedures for map generation.

The mathematical equations for different analytical models are as follows: Global Moran’s I [4], where xi is the attribute for feature I, X̅ is the mean of the corresponding attribute, wi,j is the spatial weight between feature i and j, and Si2 is the equation of the total number of features, and Getis-Ord Gi* [4], where n is the traffic crash rate value, xj is the property value for feature jth element, wi,j is the spatial weight between feature i and j, and X̅ is the mean of the variable.

## Results

**Figure 1:**
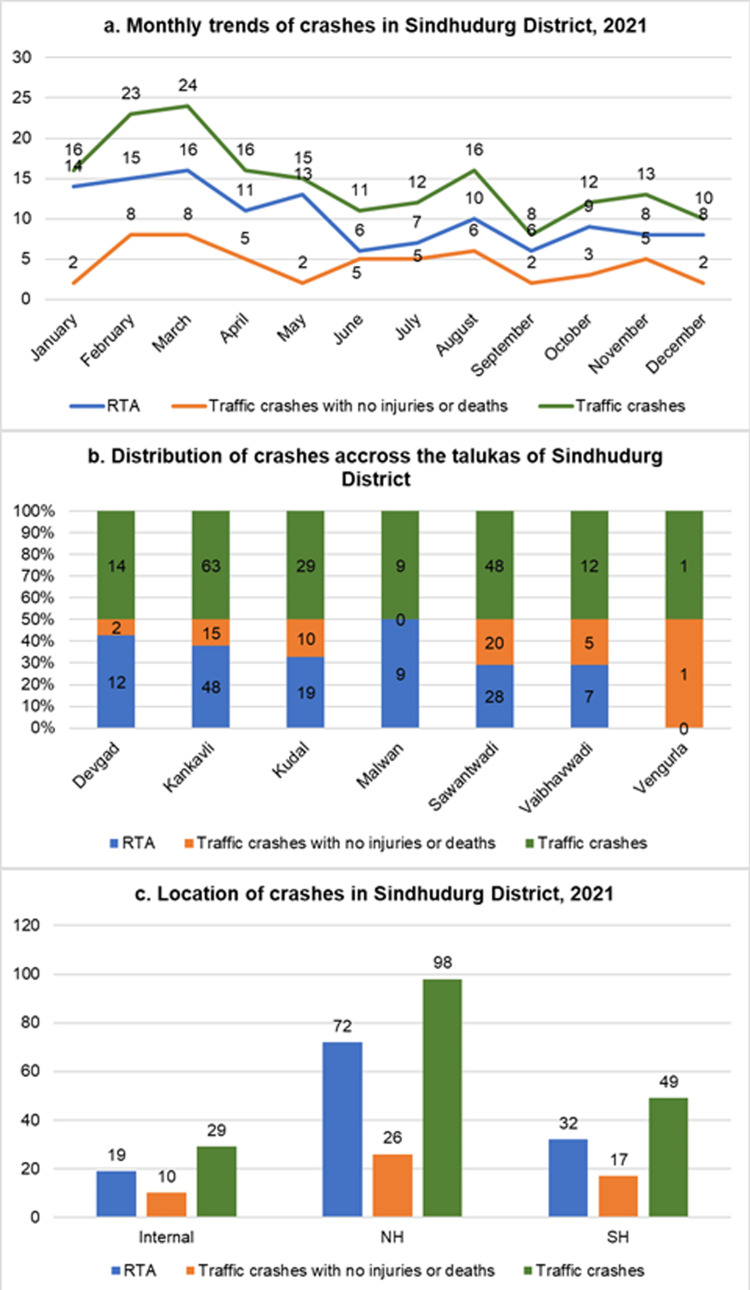
(a-c) Distribution of RTA and traffic crashes in Sindhudurg district in 2021 RTA: road traffic accident; NH: national highway; SH: state highway

The present study was conducted in Sindhudurg district and recorded 176 traffic crashes, of which 123 (69.9%) were reported as RTAs, while 53 (30.1%) resulted in no injuries or fatalities. The highest number of traffic crashes was reported in March (24, 13.6%), followed by February (23, 13.1%), while the lowest was reported in September (8, 4.5%). Among the eight talukas, the maximum traffic crash contribution was by Kankavli (63, 35.8%), followed by Sawantwadi (48, 39%), and Vengurla (1, 0.57%) had the fewest incidents, as shown in Figure 1. A chi-squared test revealed a statistically significant association between RTAs and crashes across the talukas (χ² = 13.113, p = 0.041), indicating a need for further investigation into risk factors and preventive measures.

Day crashes were found to be 118 (67%), night 37 (61%), and 21 (12%) occurred at unknown timing. Traffic crashes on weekdays (123, 70%) exceeded those on weekends (53, 30%) in the present study. Notably, most of the RTAs (72, 58.5%) occurred along the NH-66. Out of 742 villages, these roads passed through the administrative borders of 78 villages.

In 2021, 53 (30%) crashes resulted in no injuries or deaths, with the majority occurring in February and March (8, 15.1% each), predominantly in Sawantwadi (20, 37.7%) and Kankavli (15, 28.3%), along the NH-66. Of these 53, 19 (35.8%) involved vehicular collisions (Figure 1).

Overall, the crashes involved 93 heavy vehicles, 91 light vehicles, 78 two-wheelers, 24 minors, and 12 pedestrians. Among 176 crashes, 84 (47.7%) were vehicular collisions, and 92 (52.3%) were single-vehicle accidents. The collisions led to 20 deaths, of which 15 (75%) were drivers and five (25%) were passengers. The injured included 115: 53 (46.1%) drivers and 62 (53.9%) passengers. Single-vehicle accidents included 58 (63%) RTA cases, leading to 18 deaths that involved eight (44.4%) drivers, five (27.8%) passengers, and five (27.8%) pedestrians. Meanwhile, 107 injured included 27 (25.2%) drivers, 73 (68.2%) passengers, and seven (6.5%) pedestrians. It can be inferred that there was higher mortality among the drivers, while injuries were higher in the passengers.

A total of 103 RTAs resulted in 222 injuries (36 women, 162 men, 18 female children, and six male children), while 34 RTAs to 38 deaths (four women, 32 men, and two male children), yielding a fatality rate of 30.9 per 100 RTAs. It can be inferred from this that mortality and injuries were higher in men. As observed, drivers were more impacted, with 80 injured and 23 dead. Of the 15 pedestrians, 12 were affected, seven were wounded, and five were dead.

**Figure 2:**
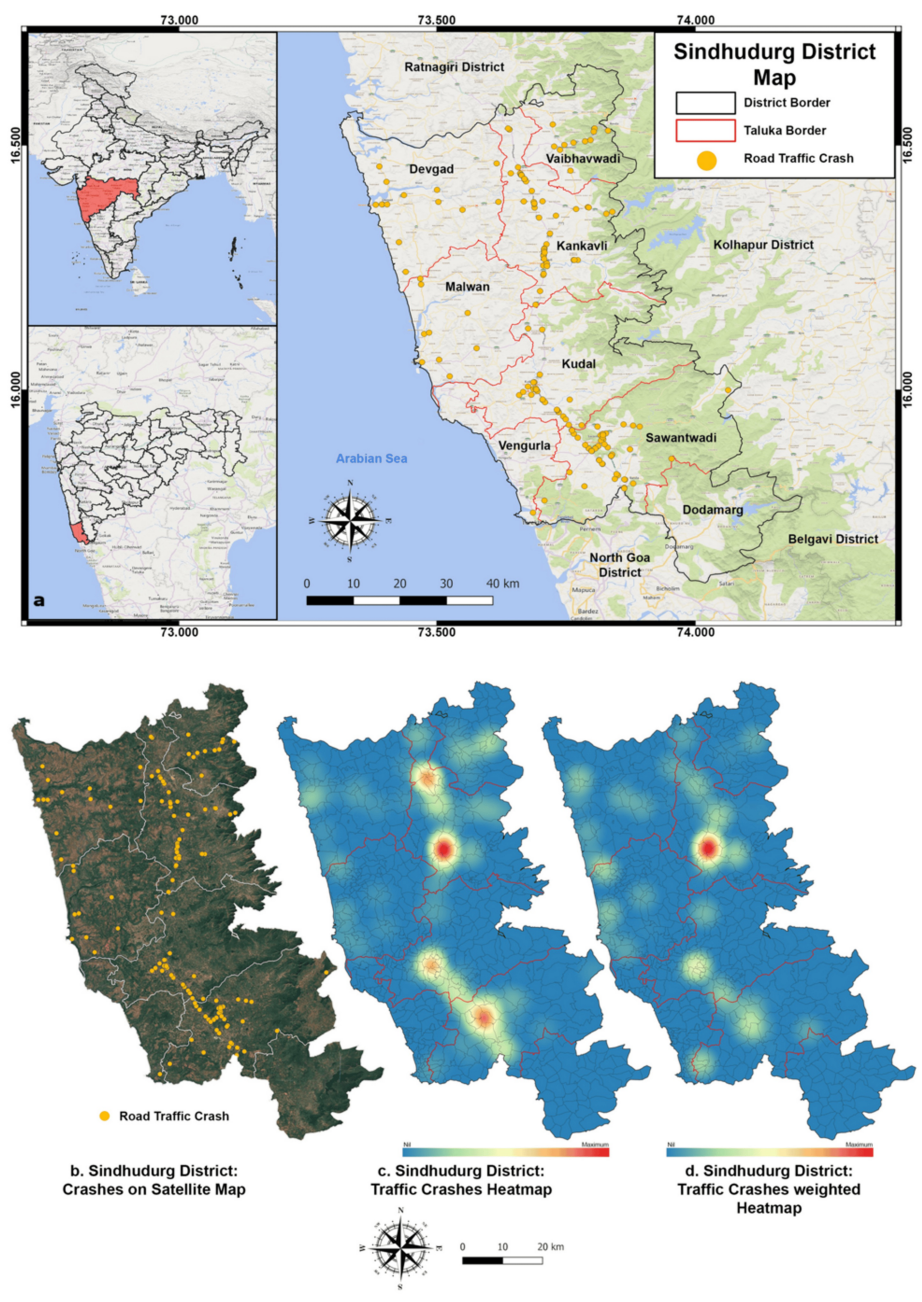
(a-d) Distribution of RTA & traffic crashes in Sindhudurg district in 2021 along with heatmaps RTA: road traffic accident

**Figure 3:**
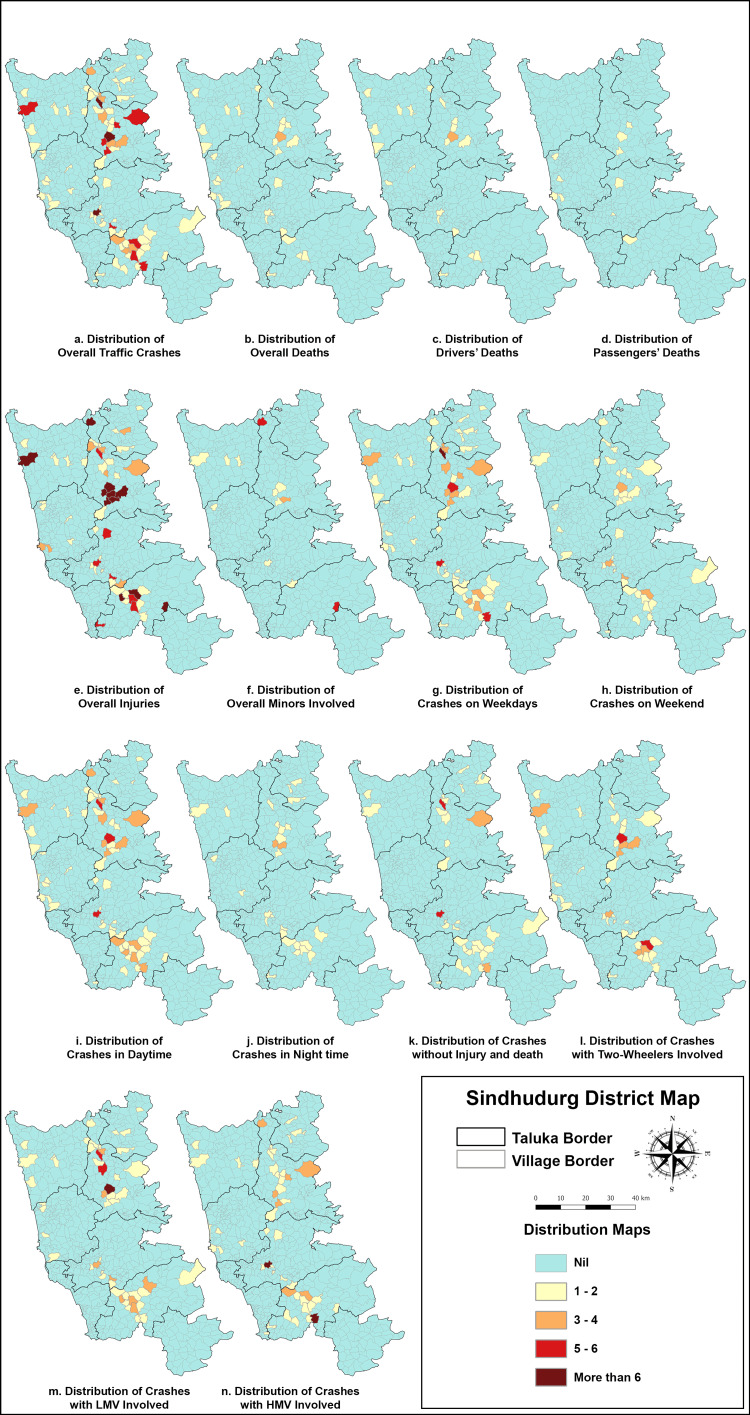
(a-n) Village-wise distribution of RTA & traffic crashes in Sindhudurg district in 2021 LMV: light motor vehicle; HMV: heavy motor vehicle; RTA: road traffic accident

GIS mapping on WGS84 in QGIS showed that most accidents occurred along NH-66, from Talere (north) to Banda (south). SH116 and NH548G also had notable crash frequencies. Most crashes occurred in low-altitude areas (<200 m), while the Western Ghat region (>200 m) witnessed fewer incidents despite daily traffic. Heatmaps identified four major clusters along NH-66, with the Janavali-Kankavli stretch showing the highest crash and fatality rates (Figures 2, 3).

Global Moran’s I analysis confirmed a statistically significant clustering pattern (p < 0.05) for accidents, injuries, deaths, and crashes without injuries, suggesting that incidents were not randomly distributed (Table 1).

**Table 1:** Spatial autocorrelation with Global Moran’s I RTA: road traffic accident; LMV: light motor vehicle; HMV: heavy motor vehicle

Input	Moran’s index	Z score	p-value	Inference
RTAs and crashes	0.152	8.802	<0.001	Significantly clustered
Deaths	0.049	2.995	0.002	Significantly clustered
Drivers’ deaths	0.052	3.196	0.001	Significantly clustered
Passengers’ deaths	-0.016	-0.901	0.367	Randomly distributed
Injuries	0.111	6.610	<0.001	Significantly clustered
Crashes involving minor	0.010	0.717	0.473	Randomly distributed
Weekday crashes	0.110	6.446	<0.001	Significantly clustered
Weekend crashes	0.115	6.697	<0.001	Significantly clustered
Day crashes	0.121	6.997	<0.001	Significantly clustered
Night crashes	0.137	8.063	<0.001	Significantly clustered
Crashes without injury and death	0.056	3.398	<0.001	Significantly clustered
Crashes involving two-wheelers	0.134	7.923	<0.001	Significantly clustered
Crashes involving LMV	0.141	8.288	<0.001	Significantly clustered
Crashes involving HMV	0.062	3.780	<0.001	Significantly clustered

**Figure 4:**
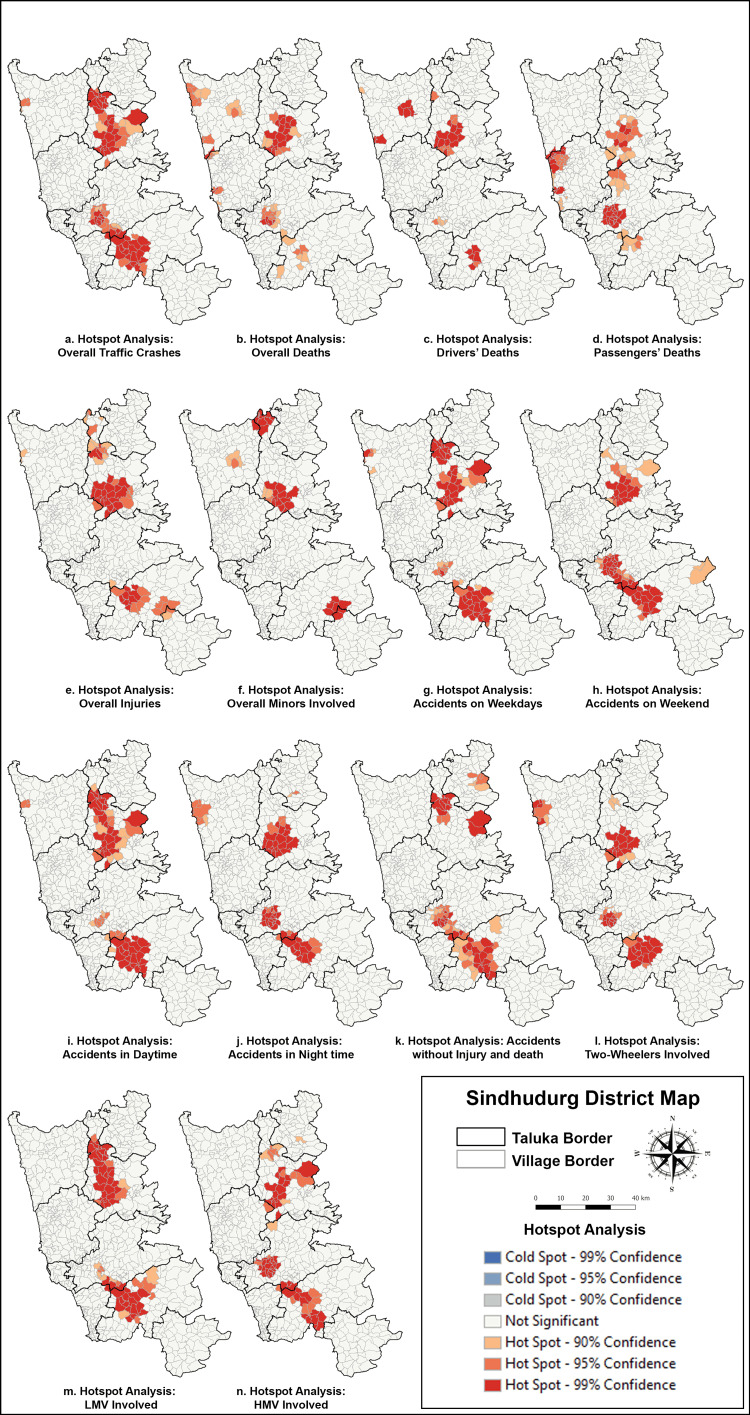
(a-n) Hotspot analysis of RTA and traffic crashes in Sindhudurg district in 2021 RTA: road traffic accident; LMV: light motor vehicle; HMV: heavy motor vehicle

Getis-Ord Gi* identified three crash hotspots, with the largest clusters in northern Sindhudurg (Talere-Kasarde to Kankavli) and southern Sindhudurg (Kudal to Banda, including Zarap-Patradevi Bypass) (Figure 4). The Janavali-Kankavli stretch was the most critical hotspot for deaths and injuries, while the Zarap-Patradevi Bypass had the highest concentration of crashes without injuries.

**Figure 5:**
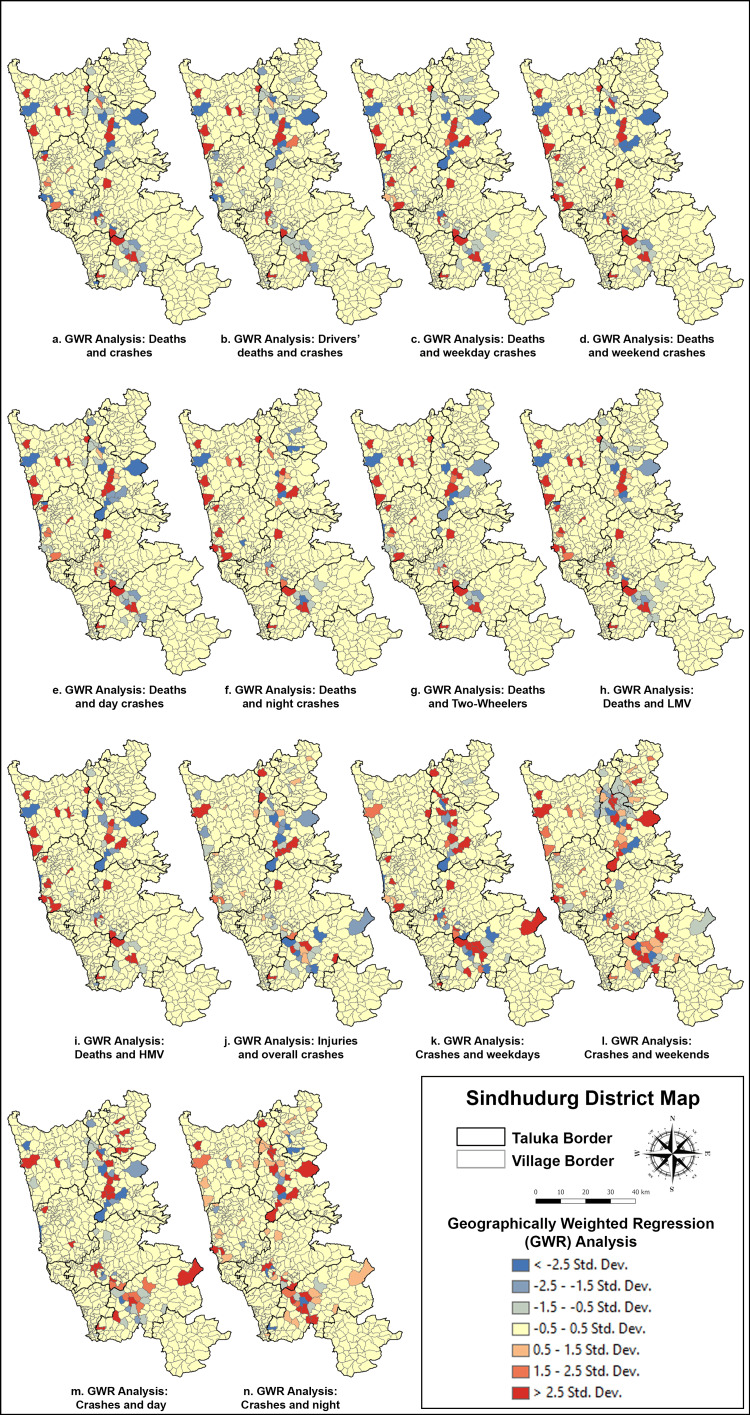
(a-n) GWR analysis of deaths and traffic crashes in Sindhudurg district in 2021 LMV: light motor vehicle; HMV: heavy motor vehicle

GWR analysis indicated that the Janavali-Kankavli stretch and Zarap Junction had the highest likelihood of fatal crashes (Figure 5). However, significance varied across the Zarap-Patradevi Bypass. Other high-risk zones for injuries included Jamsande, Talere, Humarat-Phondaghat, and the Zarap-Patradevi Bypass.

## Discussion

RTA is a serious issue and is one of the leading reasons for mortality in India [10]. According to the Stockholm Declaration, India supported the UN resolution to reduce road traffic deaths and injuries by 50% by 2030 [11]. Our study reported a fatality rate of 30.9 per 100 RTAs as compared to the 33.7 deaths per 100 accidents in India and 55.7 deaths in Maharashtra [1].

The present study's findings aligned with existing literature, identifying highways and congested regions as high-risk zones for RTAs (13-17). The predominance of crashes along NH-66, SH-116, and NH-548G highlighted the role of high-speed corridors in traffic-related morbidity and mortality. Our study highlighted how RTAs and crashes varied across talukas, particularly along the NH-66. Similar findings on geographic unit variations were reported by Mohammed et al. [4]. Our study showed a spatial dependence in crash occurrences, emphasizing the importance of targeted interventions, while they focused on model consistency across spatial units. Our discussion emphasizes real-world crash distribution and the need for location-specific road safety strategies [12]. Alizadeh et al. analyzed the spatial distribution and temporal trends of fatal intercity car traffic accidents in Iran [13]. Bisht and Tiwari analyzed fatal rear-end crashes on an Indian expressway (2012-2018) using negative binomial models [14]. They reported that rear-end crashes comprised 49% of fatalities, with trucks and cars being mostly involved. This is similar to our study but contrasts with national reports of two-wheelers being the most involved [1,14] Speed, annual average daily traffic (AADT), and vertical curve length were key risk factors, while village segments were safer [14]. Areas with entry/exit ramps on expressways and underpasses had higher crash risks [14].

Studies have linked poor traffic regulation, inadequate lighting, and high vehicle density on highways to increased accident rates [15]. Bagaria and Bagaria used spatial analysis to identify a motorbike accident hotspot that was caused by a faulty traffic light and a speed breaker. After repairs, no further incidents occurred [15].

Most crashes occurred in low-altitude areas rather than hilly roads in the Western Ghats (Figure 1), likely due to lower speeds and cautious driving in mountainous terrain. Further research on road conditions, visibility, and driver behavior in hilly sections is needed.

Seasonal trends showed peak crashes in February and March, possibly due to increased tourism, road conditions, or weather patterns. In contrast to this, some studies report higher vehicular movement and risky driving behavior before the monsoon [16].

The Janavali-Kankavli stretch is identified as a critical hotspot for fatalities and injuries, where immediate safety interventions are needed. The Zarap-Patradevi Bypass, identified as a hotspot for crashes without injuries, indicates near-miss incidents, which require road design and traffic behavior analysis to prevent future severe crashes (Figure 3).

Men were disproportionately affected in RTAs, with 162 male injuries versus 36 female injuries and 32 male deaths versus four female deaths, reflecting global trends where young male drivers are at higher risk due to greater exposure and risk-taking behavior [16]. Pedestrian casualties emphasize the need for dedicated safety measures along highways.

The study found 53 (30%) traffic crashes without injuries or fatalities, primarily in February and March along NH in Sawantwadi and Kankavli, highlighting potential future risks. A strong spatial autocorrelation (Moran’s I) of crashes, injuries, and deaths confirms that accidents cluster in high-risk areas. This supports the use of GIS-based mapping for traffic injury surveillance and intervention planning, as advocated in other studies [13].

This study provides key insights into the geographic and temporal patterns of RTAs in Sindhudurg, aiding evidence-based policy recommendations. Future research with multi-year data and risk factor analysis can further clarify accident dynamics. Strengthening speed regulation, lane discipline, and enforcement of helmet and seatbelt laws in high-risk areas is crucial. Infrastructure improvements, including better signage, rumble strips, speed cameras, and pedestrian crossings in hotspots, along with enhanced real-time monitoring on NH-66, are recommended.

## Conclusions

In Sindhudurg district, the Janavali-Kankavli stretch was identified as the most critical hotspot for fatalities and injuries, while the Zarap-Patradevi Bypass had the highest number of non-injury crashes. Spatial analysis confirmed significant clustering of crashes, deaths, and injuries. Strengthening highway safety measures and educating drivers can help reduce accident rates and improve overall traffic safety.

## Appendices

**Table 2:** Coordinates of crashes in Sindhudurg district in 2021

Latitude	Longitude	Source paper	Edition	Date	Page
16.44181	73.66214	Prahar	Sindhudurg	1/2/2021	2
15.87518	73.80096	Prahar	Sindhudurg	1/2/2021	3
15.99961	74.06554	Prahar	Sindhudurg	11/28/2021	2
15.97995	73.70538	Prahar	Sindhudurg	1/10/2021	2
16.37866	73.39435	Prahar	Sindhudurg	1/10/2021	2
16.38499	73.70533	Prahar	Sindhudurg	1/12/2021	1
15.85966	73.95572	Prahar	Sindhudurg	1/13/2021	1
15.84892	73.82171	Prahar	Sindhudurg	1/15/2021	5
15.77431	73.70906	Prahar	Sindhudurg	2/4/2021	1
16.46227	73.61628	Prahar	Sindhudurg	6/26/2021	2
16.38377	73.50324	Prahar	Sindhudurg	8/27/2021	1
15.9976	73.69526	Prahar	Sindhudurg	1/17/2021	2
16.36762	73.54998	Prahar	Sindhudurg	3/26/2021	1
16.43902	73.6635	Prahar	Sindhudurg	6/19/2021	2
15.86499	73.83857	Prahar	Sindhudurg	1/24/2021	3
15.88204	73.8084	Prahar	Sindhudurg	1/24/2021	3
16.38467	73.66903	Prahar	Sindhudurg	6/21/2021	5
15.99014	73.68357	Prahar	Sindhudurg	1/17/2021	2
16.53367	73.80589	Prahar	Sindhudurg	2/5/2021	3
16.0315	73.70008	Prahar	Sindhudurg	2/8/2021	5
16.23466	73.70674	Prahar	Sindhudurg	2/9/2021	3
16.26512	73.77355	Prahar	Sindhudurg	4/15/2021	2
16.43075	73.67027	Prahar	Sindhudurg	2/10/2021	2
15.80041	73.86391	Prahar	Sindhudurg	1/23/2021	3
15.9402	73.7503	Prahar	Sindhudurg	2/13/2021	2
16.25952	73.71242	Prahar	Sindhudurg	2/14/2021	2
15.79903	73.86401	Prahar	Sindhudurg	2/2/2021	3
15.95464	73.73669	Prahar	Sindhudurg	2/15/2021	2
15.81747	73.86429	Prahar	Sindhudurg	5/6/2021	3
15.90761	73.81791	Prahar	Sindhudurg	2/18/2021	3
15.92885	73.75625	Prahar	Sindhudurg	2/20/2021	2
15.88553	73.81354	Prahar	Sindhudurg	2/20/2021	5
16.27409	73.70715	Prahar	Sindhudurg	2/22/2021	1
15.80915	73.88123	Prahar	Sindhudurg	7/16/2021	3
15.9749	73.71014	Prahar	Sindhudurg	3/20/2021	1
15.89921	73.82301	Prahar	Sindhudurg	4/13/2021	6
15.9803	73.75816	Prahar	Sindhudurg	7/16/2021	1
16.26526	73.76579	Prahar	Sindhudurg	2/28/2021	2
16.11696	73.48523	Prahar	Sindhudurg	3/1/2021	1
15.74967	73.68683	Prahar	Sindhudurg	3/15/2021	2
15.86823	73.84036	Prahar	Sindhudurg	9/13/2021	3
16.29732	73.71298	Prahar	Sindhudurg	3/5/2021	5
16.02785	73.52516	Prahar	Sindhudurg	3/14/2021	1
15.86542	73.83855	Prahar	Sindhudurg	11/30/2021	3
16.42813	73.67177	Prahar	Sindhudurg	3/16/2021	3
16.43816	73.66459	Prahar	Sindhudurg	4/15/2021	2
16.28748	73.70837	Prahar	Sindhudurg	3/18/2021	3
15.84962	73.82105	Prahar	Sindhudurg	3/19/2021	2
16.43816	73.66459	Prahar	Sindhudurg	6/26/2021	2
16.2528	73.71394	Prahar	Sindhudurg	3/20/2021	1
16.06184	73.50463	Prahar	Sindhudurg	3/20/2021	2
16.43816	73.66459	Prahar	Sindhudurg	7/9/2021	2
16.43816	73.66459	Prahar	Sindhudurg	7/21/2021	2
16.00092	73.69309	Prahar	Sindhudurg	3/21/2021	5
16.27356	73.70726	Prahar	Sindhudurg	3/22/2021	2
16.1254	73.67736	Prahar	Sindhudurg	3/24/2021	2
16.53156	73.64101	Prahar	Sindhudurg	3/26/2021	1
16.31929	73.71965	Prahar	Sindhudurg	9/4/2021	2
16.25952	73.71242	Prahar	Sindhudurg	3/26/2021	2
15.81705	73.86536	Prahar	Sindhudurg	3/26/2021	2
15.88069	73.79515	Prahar	Sindhudurg	3/27/2021	3
16.43816	73.66459	Prahar	Sindhudurg	8/4/2021	1
16.00185	73.68771	Prahar	Sindhudurg	4/1/2021	3
16.25374	73.70665	Prahar	Sindhudurg	4/2/2021	1
16.35947	73.82912	Prahar	Sindhudurg	4/3/2021	6
15.92512	73.81196	Prahar	Sindhudurg	4/7/2021	3
16.53156	73.64101	Prahar	Sindhudurg	4/11/2021	5
16.372	73.68994	Prahar	Sindhudurg	4/13/2021	5
15.95962	73.73335	Prahar	Sindhudurg	11/15/2021	5
16.40628	73.68442	Prahar	Sindhudurg	8/19/2021	3
15.9597	73.73316	Prahar	Sindhudurg	11/23/2021	6
16.50734	73.78783	Prahar	Sindhudurg	3/3/2021	3
16.28867	73.709	Prahar	Sindhudurg	5/4/2021	2
16.40835	73.50061	Prahar	Sindhudurg	4/16/2021	3
16.53368	73.80634	Prahar	Sindhudurg	4/21/2021	1
16.37792	73.38167	Prahar	Sindhudurg	4/21/2021	2
16.39766	73.43645	Prahar	Sindhudurg	1/4/2021	2
16.20179	73.70048	Prahar	Sindhudurg	4/23/2021	2
16.27406	73.70716	Prahar	Sindhudurg	2/24/2021	5
16.28393	73.70849	Prahar	Sindhudurg	6/17/2021	2
16.52922	73.8324	Prahar	Sindhudurg	10/20/2021	3
16.37949	73.68933	Prahar	Sindhudurg	5/7/2021	3
15.98944	73.65989	Prahar	Sindhudurg	5/9/2021	3
16.50983	73.79841	Prahar	Sindhudurg	5/11/2021	3
16.5001	73.74771	Prahar	Sindhudurg	5/13/2021	3
15.99611	73.66775	Prahar	Sindhudurg	5/16/2021	2
15.91777	73.76221	Prahar	Sindhudurg	5/16/2021	3
16.12322	73.70468	Prahar	Sindhudurg	5/17/2021	1
16.00305	73.69227	Prahar	Sindhudurg	5/21/2021	2
15.91142	73.8284	Prahar	Sindhudurg	5/23/2021	3
16.44181	73.66214	Prahar	Sindhudurg	5/25/2021	2
16.2528	73.71394	Prahar	Sindhudurg	5/27/2021	2
16.50466	73.76533	Prahar	Sindhudurg	5/27/2021	3
16.52708	73.80466	Prahar	Sindhudurg	12/26/2021	2
16.27594	73.70746	Prahar	Sindhudurg	6/13/2021	3
16.28322	73.70856	Prahar	Sindhudurg	11/30/2021	1
16.00092	73.69309	Prahar	Sindhudurg	3/20/2021	3
16.00692	73.67699	Prahar	Sindhudurg	7/28/2021	2
16.01573	73.68941	Prahar	Sindhudurg	8/5/2021	2
15.92803	73.78123	Prahar	Sindhudurg	6/25/2021	3
16.01562	73.68768	Prahar	Sindhudurg	8/12/2021	3
16.27542	73.70731	Prahar	Sindhudurg	7/15/2021	1
16.43075	73.67027	Prahar	Sindhudurg	6/29/2021	2
16.27424	73.70715	Prahar	Sindhudurg	8/3/2021	1
16.01562	73.68768	Prahar	Sindhudurg	10/24/2021	3
16.49682	73.72789	Prahar	Sindhudurg	7/2/2021	3
15.91049	73.82221	Prahar	Sindhudurg	7/5/2021	2
16.22706	73.46813	Prahar	Sindhudurg	7/6/2021	2
15.88039	73.80755	Prahar	Sindhudurg	2/28/2021	2
15.86572	73.83854	Prahar	Sindhudurg	1/16/2021	2
15.95853	73.73405	Prahar	Sindhudurg	7/11/2021	2
16.00092	73.69309	Prahar	Sindhudurg	12/15/2021	1
16.5418	73.72524	Prahar	Sindhudurg	8/7/2021	2
15.92518	73.8939	Prahar	Sindhudurg	3/17/2021	2
16.42555	73.67333	Prahar	Sindhudurg	7/17/2021	2
15.92735	73.88102	Prahar	Sindhudurg	4/15/2021	3
15.88265	73.83191	Prahar	Sindhudurg	3/21/2021	2
16.15715	73.56007	Prahar	Sindhudurg	1/20/2021	2
15.88069	73.79515	Prahar	Sindhudurg	6/30/2021	3
16.38362	73.68833	Prahar	Sindhudurg	2/23/2021	2
16.38058	73.68924	Prahar	Sindhudurg	8/28/2021	2
16.53378	73.63843	Prahar	Sindhudurg	8/8/2021	3
16.25828	73.71361	Prahar	Sindhudurg	8/12/2021	2
16.48988	73.73921	Prahar	Sindhudurg	8/12/2021	3
15.9107	73.76584	Prahar	Sindhudurg	4/21/2021	3
15.91752	73.76249	Prahar	Sindhudurg	8/12/2021	3
16.05676	73.4715	Prahar	Sindhudurg	8/14/2021	1
15.87544	73.80032	Prahar	Sindhudurg	8/18/2021	2
16.30148	73.42668	Prahar	Sindhudurg	12/18/2021	1
16.35152	73.69858	Prahar	Sindhudurg	8/23/2021	2
16.24101	73.43981	Prahar	Sindhudurg	7/9/2021	5
15.90255	73.77368	Prahar	Sindhudurg	10/17/2021	3
16.08509	73.57734	Prahar	Sindhudurg	9/3/2021	5
16.36916	73.77049	Prahar	Sindhudurg	9/4/2021	2
16.1143	73.47563	Prahar	Sindhudurg	2/10/2021	1
16.38376	73.61971	Prahar	Sindhudurg	9/6/2021	2
15.89678	73.81889	Prahar	Sindhudurg	9/6/2021	3
16.23679	73.70757	Prahar	Sindhudurg	9/13/2021	3
15.83201	73.75763	Prahar	Sindhudurg	4/29/2021	2
16.17403	73.69197	Prahar	Sindhudurg	3/3/2021	2
16.44749	73.75958	Prahar	Sindhudurg	9/27/2021	2
16.21536	73.4697	Prahar	Sindhudurg	10/5/2021	1
15.87528	73.8005	Prahar	Sindhudurg	10/5/2021	2
16.28361	73.70846	Prahar	Sindhudurg	10/6/2021	3
16.45456	73.65789	Prahar	Sindhudurg	10/17/2021	3
15.87886	73.87506	Prahar	Sindhudurg	2/25/2021	3
16.2528	73.71394	Prahar	Sindhudurg	10/18/2021	3
15.80344	73.78669	Prahar	Sindhudurg	6/20/2021	3
16.37865	73.39368	Prahar	Sindhudurg	10/21/2021	2
16.4421	73.64379	Prahar	Sindhudurg	10/23/2021	2
16.35565	73.73136	Prahar	Sindhudurg	10/24/2021	1
16.37305	73.79315	Prahar	Sindhudurg	5/31/2021	5
16.00085	73.69293	Prahar	Sindhudurg	11/1/2021	2
16.37856	73.40369	Prahar	Sindhudurg	11/3/2021	1
15.88914	73.82547	Prahar	Sindhudurg	11/5/2021	3
16.26731	73.7085	Prahar	Sindhudurg	11/5/2021	3
16.26521	73.7671	Prahar	Sindhudurg	11/9/2021	2
16.43075	73.67027	Prahar	Sindhudurg	11/10/2021	3
16.37235	73.79504	Prahar	Sindhudurg	9/21/2021	2
15.85671	73.81526	Prahar	Sindhudurg	11/17/2021	3
16.36356	73.8407	Prahar	Sindhudurg	12/3/2021	3
16.28748	73.70837	Prahar	Sindhudurg	11/22/2021	3
15.82594	73.85171	Prahar	Sindhudurg	2/10/2021	3
15.8184	73.84561	Prahar	Sindhudurg	2/14/2021	2
15.85707	73.81507	Prahar	Sindhudurg	6/29/2021	3
15.9807	73.70529	Prahar	Sindhudurg	11/20/2021	1
16.43075	73.67027	Prahar	Sindhudurg	3/30/2021	2
15.90364	73.82224	Prahar	Sindhudurg	12/8/2021	3
15.88758	73.78827	Prahar	Sindhudurg	12/12/2021	2
15.90805	73.82265	Prahar	Sindhudurg	12/12/2021	3
15.82834	73.84896	Prahar	Sindhudurg	12/14/2021	3
16.42477	73.40249	Prahar	Sindhudurg	4/16/2021	1
15.94601	73.7455	Prahar	Sindhudurg	1/17/2021	2
16.28048	73.70934	Prahar	Sindhudurg	12/19/2021	2
15.92977	73.86257	Prahar	Sindhudurg	12/24/2021	2
16.45612	73.38867	Prahar	Sindhudurg	2/16/2021	1
